# Study of Total Ammoniacal Nitrogen Recovery Using Polymeric Thin-Film Composite Membranes for Continuous Operation of a Hybrid Membrane System

**DOI:** 10.3390/polym17121696

**Published:** 2025-06-18

**Authors:** Shirin Shahgodari, Joan Llorens, Jordi Labanda

**Affiliations:** Department of Chemical Engineering and Analytical Chemistry, University of Barcelona, Martí i Franquès 1, 08028 Barcelona, Spain

**Keywords:** ammonium recovery, membrane technology, nanofiltration, hybrid membrane system

## Abstract

This study examined total ammoniacal nitrogen (TAN) rejection by two reverse osmosis (RO) and two nanofiltration (NF) membranes as a function of pH for three ammonium salts to optimize conditions for a hybrid membrane system that can produce high-purity TAN streams suitable for reuse. The results showed that TAN rejection was significantly influenced by membrane type, feed pH, and the ammonium salt used. This study represents the first attempt to simulate real manure wastewater conditions typically found in pig manure. TAN rejection for (NH_4_)_2_SO_4_ and NH_4_HCO_3_ reached up to 95% at pH values below 7, with the SW30 membrane showing the highest performance (99.5%), attributed to effective size exclusion and electrostatic repulsion of SO_4_^2−^ and HCO_3_^−^ ions. In contrast, lower rejection was observed for NH_4_Cl, particularly with the MPF-34 membrane, due to its higher molecular weight cut-off (MWCO), which diminishes both exclusion mechanisms. TAN rejection decreased markedly with increasing pH across the BW30, NF90, and MPF-34 membranes as the proportion of uncharged NH_3_ increased. The lowest rejection rates (<15%) were recorded at pH 11.5 for both NF membranes. These results reveal a notable shift in separation behavior, where NH_3_ permeation under alkaline conditions becomes dominant over the commonly reported NH_4_^+^ retention at low pH. This novel insight offers a new perspective for optimizing membrane-based ammonia recovery in systems simulating realistic manure wastewater conditions. TAN recovery was evaluated using a hybrid membrane system, where NF membranes operated at high pH promoted NH_3_ permeation, and the SW30 membrane at pH 6.5 enabled TAN rejection as (NH_4_)_2_SO_4_. This hybrid system insight offers a new perspective for optimizing membrane-based ammonia recovery in systems simulating realistic manure wastewater conditions. Based on NH_3_ permeation and membrane characteristics, the NF90 membrane was operated at pH 9.5, achieving a TAN recovery of 48.3%, with a TAN concentration of 11.7 g/L, corresponding to 0.9% nitrogen. In contrast, the MPF-34 membrane was operated at pH 11.5. The NF90–SW30 system also achieved a TAN recovery of 48.3%, yielding 11.7 g/L of TAN with a nitrogen content of 1.22%. These nitrogen concentrations indicate that both retentate streams are suitable for use as liquid fertilizers in the form of (NH_4_)_2_SO_4_. A preliminary economic assessment estimated the chemical consumption cost at 0.586 EUR/kg and 0.729 EUR/kg of (NH_4_)_2_SO_4_ produced for the NF90–SW30 and MPF-34–SW30 systems, respectively.

## 1. Introduction

Livestock farming produces large amounts of waste that can harm the environment if not managed properly. Liquid manure, a mix of solid and liquid excrement, often causes contamination issues [1]. However, manure also contains valuable nutrients like nitrogen, phosphorus, and potassium [2]. Proper treatment of livestock waste can recover these nutrients, reducing environmental impact. This process also contributes to the conservation of natural resources by reducing nutrient loss [3].

The predominant form of nitrogen in livestock manure is ammonium ions, although urea and amino acids are also found in smaller amounts due to rapid enzymatic urea hydrolysis [4]. For instance, pig manure contains high concentrations of total ammoniacal nitrogen (TAN), with values higher than 2000 mg/L [2]. Consequently, both primary and digested livestock manure have potential for TAN recovery, with the objective of obtaining a product rich in nutrients that can be reused as a fertilizer.

Several studies have focused on the removal of TAN from wastewater. Among the methods employed, chemical processes such as chlorination and electrochemical oxidation play a significant role in eliminating TAN [5,6]. Biological processes are also employed, where ammonium is enzymatically converted into nitrogen gas [7,8]. This biological transformation reduces TAN levels effectively. In addition to biological and chemical approaches, physical processes have been used for TAN recovery rather than removal. Classical techniques include ammonia stripping, adsorption, ion-exchange, and precipitation as struvite [9,10,11]. Membrane technology has been also used to remove and recover TAN. At pH values lower than the pKa of the NH_4_^+^-NH_3_ acid–base equilibrium, NH_4_^+^ is the predominant specie that can be effectively retained by membranes due to the formation of an ionic interaction with large anions such as sulfate or hydrogen carbonate [12,13]. NH_3_ can permeate freely through the membrane matrix to be recovered in the permeate stream when the manure pH is above the pKa [14].

Energy-efficient membrane technology has been used to recover TAN, such as gas permeation contactors and forward osmosis. In both processes, the transfer of the molecules through the membrane wall is controlled by the chemical concentration gradient between the two phases or solutions in contact with the membrane [15,16]. The hydrophobic membranes for gas permeation prevent the permeation of the water molecules and allow NH_3_ diffusion into a trapping acidic solution that then converts ammonia into NH_4_^+^ [17]. Forward osmosis is mainly used to concentrate the feed solution, enabling water molecules to permeate through the membrane due to the osmotic pressure difference between the feed and the draw solutions. Similarly, NH_3_, as a non-charged molecule, can also diffuse through the forward osmosis membranes at a basic pH for recovery into the draw solutions [18]. Gas permeation contactors and forward osmosis have the limitation of being slow processes where the permeate solution presents low concentrations of the recovered TAN. Moreover, the reconcentration of the draw solution in forward osmosis increases operational costs [19].

Pressure-driven membrane technologies, including reverse osmosis (RO) and nanofiltration (NF), can also be employed for TAN removal and recovery. Some studies have concentrated TAN to produce a nutrient-rich slurry, recovering NH_4_^+^ in the retentate stream while simultaneously obtaining purified water for reuse in permeate stream. Thörneby et al. (1999) achieved a volume reduction of approximately 60% in the liquid fraction of pig slurry and a remarkable 93% removal of NH_4_^+^ at a pH of around 7 [20]. Both RO and NF processes exhibit a significant rejection of small charged solutes, particularly inorganic salts, which occurs mainly due to the electrostatic repulsion between the solute and the membrane surface charges driven by the Donnan potential [21,22]. Using an RO membrane, Shin et al. (2021) [10] assessed the effluent from an anaerobic membrane bioreactor with a TAN feed concentration of 50 mg/L. They observed a peak NH_4_^+^ rejection of 99% at pH 6, which gradually decreased to 96% and 94% at pH 4 and 8, respectively. Masse et al. (2008) [23] studied the TAN rejection of pretreated swine manure at different pH levels. They observed that acidification significantly improved TAN rejection, which reached up to 98%. Additionally, NF membranes exhibited lower TAN rejections, which fell below 80% at pH values lower than 8 [24]. Nevertheless, the rejection of ammonia has received less attention, especially at pH levels higher than the pKa. Recent research works have explored NH_3_ recovery from hydrolyzed human urine at pH levels of up to 11, with NF membranes showing promising results of up to 90% recovery [25,26].

The aim of this study was to evaluate TAN recovery using commercial RO and NF membranes to determine optimal operating conditions for a hybrid membrane system capable of producing a TAN stream suitable for reuse as fertilizer. The investigation focused on TAN rejection at concentrations representative of real livestock manure, examining the effect of feed pH using three ammonium salts: NH_4_Cl, (NH_4_)_2_SO_4_, and NH_4_HCO_3_. In contrast to most existing studies that focus on NH_4_^+^ retention in the retentate, this work prioritized enhancing NH_3_ permeation at alkaline pH to improve TAN recovery efficiency. The influence of organic matter was assessed to simulate more realistic manure wastewater conditions and evaluate its impact on membrane performance. Additionally, the study investigates, for the first time, a hybrid NF–RO membrane system as a practical and scalable solution for the simultaneous recovery of TAN and clean water from high-strength manure wastewater. The proposed system operates under a two-stage configuration: (i) the first stage employs NF membranes under alkaline conditions to promote TAN permeation in the form of NH_3_, a process not extensively explored in the literature; (ii) the second stage uses RO membranes under acidic conditions to concentrate the recovered TAN as (NH_4_)_2_SO_4_, a valuable liquid fertilizer. This novel approach offers a new pathway for nutrient recovery that aligns with circular economy principles.

## 2. Materials and Methods

### 2.1. Feed Solutions and Membranes

Three different ammonium salts were used to evaluate TAN recovery with NF membranes (NH_4_Cl, (NH_4_)_2_SO_4_, and NH_4_HCO_3_), which were supplied by Panreac Química S.L.U. (Barcelona, Spain) in the pure grade. Single-solute solutions were prepared with a fixed osmotic pressure of the feed solution of around 9 bar, which was achieved with 0.2 M NH_4_Cl and NH_4_HCO_3_ and 0.175 M (NH_4_)_2_SO_4_. The osmotic pressure of the three solutions was calculated using the OLI Stream Analyzer software v12 (OLI Systems, Inc., Parsippany, NJ, USA), which is based on the Helgeson–Kirkham–Flowers (HKF) thermodynamic model [27] that enables osmotic pressure calculations considering the non-ideality of solutions. These concentrations reflect the typical high concentrations of TAN in wastewater, such as pig manure, with TAN contents of 3.6 g/L for NH_4_Cl and NH_4_HCO_3_ and 6.3 g/L for (NH_4_)_2_SO_4_ [28]. Pure water, sourced from the Milli-Q water purification system, was filtered through a membrane with a pore diameter of 45 μm. The feed pH was adjusted by adding small amounts of concentrated acid solution (HCl for NH_4_Cl and NH_4_HCO_3_ solutions and H_2_SO_4_ for (NH_4_)_2_SO_4_ solutions) or concentrated NaOH solutions for a basic pH. Additionally, glucose and glycine were added to some feed solutions to evaluate their effect on membrane performance. These organic solutes were supplied by Sigma-Aldrich (Madrid, Spain) and were obtained in a pure grade. Initially, membrane performance was assessed using a feed solution of 0.2 M NaCl (also of pure grade) from Panreac Química S.L.U. (Barcelona, Spain).

This study utilized four thin-film composite polymeric membranes: two RO membranes (SW30 and BW30) and two NF membranes (NF90 and SelRO^®^ MPF-34). The SW30, BW30, and NF90 membranes (supplied by DuPont Barcelona, Spain) feature a fully aromatic polyamide top layer and can operate at temperatures of up to 90 °C and in a pH range of 2–11 (Table 1). By contrast, the MPF-34 membrane (supplied by Koch Membrane Systems, MA, USA) is a proprietary thin-film composite with a maximum operating temperature of 70 °C and a functional pH range of 0–14. The primary differences in the performance of the four membranes are attributed to their molecular weight cut-off (MWCO), which directly influences both water permeability and salt rejection, as shown in Table 1. Another key distinction is the isoelectric point (IEP) of the membranes: while the polyamide membranes exhibit similar IEP values, the MPF-34 membrane has an IEP closer to neutral pH. To evaluate the difference in chemical composition between polyamide membrane (such as NF90) and the MPF-34 membrane, we have conducted an attenuated total reflectance Fourier transform infrared spectroscopy (ATR-FTIR) of the two membranes. The ATR-FTIR spectrum was recorded between 4000 and 525 cm^−1^ at 4 cm^−1^ resolutions at the transmittance mode and after 32 scans.

### 2.2. Experimental Setup

The experimental setup included a reservoir tank, a chiller, a pump, pressure gauges, a filtration cell, and flow meters for the retentate and permeate. The laboratory-scale SEPA CF II membrane element cell (Osmonics, Minnetonka, MN, USA) was used, which operates in the cross-flow mode and has an effective membrane area of 140 cm^2^. The feed chamber was a rectangular channel measuring 14.5 cm in length, 9.5 cm in width, and 0.43 mm in height. It contained a woven spacer composed of cylindrical filaments arranged in a diamond configuration, with crossing filaments forming a 90° angle. This setup resulted in a channel porosity of 0.92 [30] and a hydraulic diameter of 0.50 mm, which was calculated using the procedure described by Drazevic et al. [31]. The feed flow was introduced into the membrane cell using a positive displacement pump, supplied by CAT-PUMPS (Kontich, Belgium), which featured a closed-pipe pressure dampener to minimize pressure fluctuations. The pump was also equipped with a variable frequency drive to fix the required feed flow, ensuring it circulated tangentially to the membrane at a fixed crossflow velocity of 1.5 m/s. The feed tank was connected to a chiller to maintain a constant temperature of 25 ± 0.1 °C for all experiments.

### 2.3. Filtration Procedure

The membranes were first conditioned with demineralized water at room temperature for 24 h, prior to installation in the membrane cell. The membranes were then compacted by filtering pure water at a constant pressure of 20 bar for 2 h, during which the permeate flow was measured to ensure consistency. All filtration runs were conducted in the steady-state mode, with both the concentrate and permeate streams being recycled back to the feed tank. The feed solution was circulated through the membrane for 2 h to reach steady-state TAN concentrations at a constant pressure of 20 bar. At this point, the permeate flow was measured and samples of the concentrate and permeate streams were collected to determine TAN content, conductivity, and pH. All experiments were conducted in duplicate, with new feed solutions prepared for each pH value. Filtration began with a feed pH of 5 and proceeded with solutions of increasing pH values. New membrane coupons were used for each ammonium salt solution.

The salt concentration was calculated by measuring conductivity using the HQ40d meter (Hach, Loveland, CO, USA) integrated with an IntelliCAL™ CDC401 Laboratory 4-Poles Graphite Conductivity Cell. The pH of the solution was determined from the IntelliCAL™ CDC and PHC probes connected to an HQ40d multimeter (Hach, Loveland, CO, USA). The TAN content was determined using a high-performance ammonium ion-selective electrode Orion 9512HPBNWP (Thermo Fisher Scientific Inc., Waltham, MA, USA), following the 4500-NH3D procedure. Glucose and glycine concentrations were determined as total organic carbon (TOC) using an ASI-V Shimadzu analyzer (Shimadzu, Columbia, MD, USA). All measurements were conducted in triplicate to ensure reproducibility, with mean values calculated at an alpha level of 0.05 for statistical significance.

The permeate flux, $J_{v}$ (L/(m^2^·h)), and solute rejection, $R_{s}$ (%), were calculated based on the permeate flow, *P* (L/h), and solute concentrations as follows:

(1) $$J_{v}=\frac{P}{A_{m}}$$

(2) $$R_{s}=1-\frac{C_{s,P}}{C_{s,R}}$$

where $A_{m}$ (m^2^) is the effective membrane area, and $C_{s,R}$ (mol/L) and $C_{s,P}$ (mol/L) are the solute concentration in the retentate and permeate streams, respectively.

## 3. Results and Discussion

### 3.1. Membrane Performance

**Figure 1:**
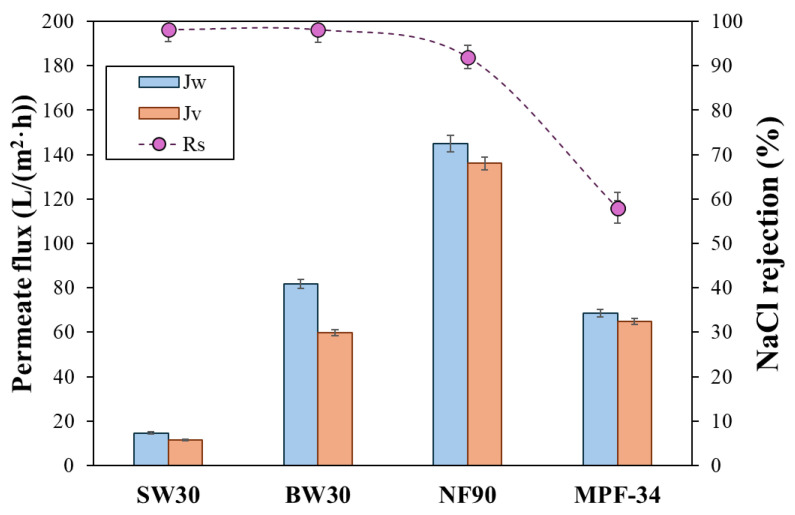
Pure water permeate flux, *J_w_*, and permeate flux, *J_v_*, as well as NaCl rejection, *R_s_*, for the feed solution of 0.1 M NaCl with the four membranes. The operating pressure was 20 bar. The error bars were calculated with a 95% confidence limit.

The performance of the four membranes was evaluated by determining the permeate flux and NaCl rejection using both pure water and a feed solution of 0.1 M NaCl, all while operating at a constant pressure of 20 bar. Figure 1 shows the pure water permeate flux, permeate flux, and NaCl rejection. As expected, the permeate flux was higher for pure water than the NaCl solution for all membranes. The SW30 membrane, being an RO membrane, showed the lowest pure water permeate flux at 14.6 L/(m^2^·h) and an even lower flux of 11.4 L/(m^2^·h) for the NaCl solution. In contrast, the NF90 membrane exhibited the highest permeate flux, reaching 145 L/(m^2^·h) with pure water and 136 L/(m^2^·h) with the NaCl solution, indicating higher water permeability under these conditions. The BW30 and MPF-34 membranes showed intermediate performance, with pure water fluxes of 81.6 L/(m^2^·h) and 68.6 L/(m^2^·h), respectively.

**Figure 2:**
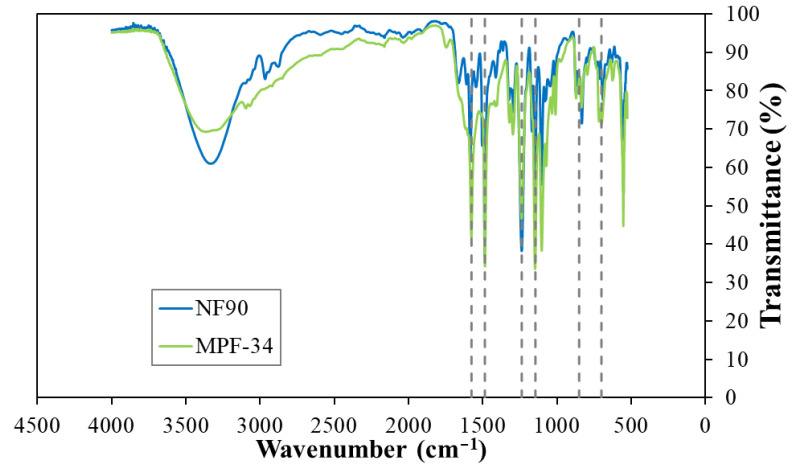
ATR-FTIR spectra of the NF90 and MPF-34 membranes, with dashed lines indicating the characteristic peaks corresponding to pure poly(p-phenylene ether sulfone).

ATR-FTIR spectroscopy was performed to investigate differences in the chemical composition of the NF membranes (Figure 2). The spectra of both membranes exhibited highly similar profiles, with identical peak positions, indicating comparable surface chemistry. Comparison with the spectrum of pure poly(p-phenylene ether sulfone) revealed a complete overlap of characteristic peaks across all three spectra. This suggests that the observed signals result from a superposition of the active and intermediate layers, both typically containing poly(ether sulfone) groups. Given the relative thickness of the layers, it is likely that the dominant contribution to the spectra originates from the intermediate layer [29,32].

The commercial membranes used in this study have been extensively characterized in the literature using conventional techniques to assess their physical properties. For instance, small-angle neutron scattering (SANS) has been applied to estimate the surface roughness of the active layer, with root-mean-squared (RMS) values reported between 60 and 65 nm for the NF90 membrane. Contact angle measurements for this membrane range from 60° to 68°, indicating moderate hydrophilicity. Cross-sectional SEM imaging has also been used to determine membrane thickness, with values between 180 and 300 nm for NF90. In contrast, the MPF-34 membrane exhibits markedly different characteristics, including a lower surface roughness (5–10 nm), lower contact angles (39–50°), and a significantly thicker active layer, approximately 1 µm [32,33]. Although a lower contact angle generally implies higher hydrophilicity and potentially greater water permeability, this is not always the case. For MPF-34, the reduced water flux (despite its hydrophilic surface) can be attributed to the membrane’s increased thickness and smoother surface, both of which influence permeation behavior. Therefore, the most significant difference between the two NF membranes (apart from the unknown chemical composition of the MPF-34 active layer) is the much greater thickness of its active layer. This structural feature aligns with the lower water flux observed for MPF-34 compared to NF90 (Figure 1), as discussed earlier.

In the presence of the NaCl solution, the permeate flux decreased to 59.80 L/(m^2^·h) for BW30 and 64.9 L/(m^2^·h) for MPF-34. These findings are consistent with previously reported results in the literature. For instance, Ricci et al. [34] obtained a permeate flux of 58 L/(m^2^·h) at 20 bar and 20 °C for the MPF-34 membrane, illustrating the impact of the solute presence on reducing flux rates, likely due to both osmotic pressure differences and membrane–solute interactions [35]. The influence of NaCl was more relevant for the BW30 membrane, which displayed a higher solute rejection. These results align with the values reported by the manufacturer, even though the experimental conditions varied between the membranes.

Regarding NaCl rejection (depicted by pink circles in Figure 1), the results showed significant variation among the membranes due to the differences in pore size and membrane surface properties. The SW30 and BW30 membranes showed the highest NaCl rejection, with values of up to 98%. The NF90 membrane, while exhibiting the highest permeate flux rates, showed a slightly reduced NaCl rejection at 92%. This is the typical behavior of NF membranes that aligns with the well-established mechanisms of NF membranes, particularly size exclusion and Donnan (electrostatic) exclusion effects [36,37]. At neutral pH, the polyamide active layers of the NF90 membrane acquire a negative surface charge due to the deprotonation of carboxyl and amide groups. This negative charge enhances the rejection of anions such as Cl^−^ via electrostatic repulsion, thereby contributing to increased NaCl rejection [29]. The importance of the two partitioning equilibria is evidenced by the performance of the NF270 membrane, which, despite having a larger MWCO than the NF90 membrane, still exhibits a NaCl rejection of approximately 50% [38]. In contrast, the MPF-34 membrane showed a lower NaCl rejection of around 60%, even though it has a permeate flux comparable to that of the BW30 membrane. The relatively lower salt rejection of the MPF-34 membrane can be primarily attributed to its larger pore size, which facilitates the transport of NaCl [39]. Additionally, the IEP of the MPF-34 membrane is approximately pH 6. At neutral pH, the membrane surface is only weakly negatively charged, resulting in reduced electrostatic repulsion toward chloride ions compared to membranes like NF90, which possess a more negatively charged surface [22]. This reduced repulsion further contributes to the lower NaCl rejection observed for the MPF-34 membrane.

### 3.2. Filtration of Ammonium Salts with RO and NF Membranes

#### 3.2.1. TAN Rejection

The study aimed to evaluate the performance of the SW30, BW30, NF90, and MPF-34 membranes in the rejection of TAN under a constant operating pressure of 20 bar while varying the pH of the feed solution. The rejection of solutes is an important factor when determining the effectiveness of the membranes in separating solutes from water, such as in desalination or water purification. In these applications, high solute rejection is typically preferred to prevent salts from passing through the membrane and to retain them in the retentate stream. However, in the context of TAN recovery, the goals differ. Instead of maximizing rejection, the aim is to ensure more TAN content passes through the membrane into the permeate while retaining salts in the retentate. This would produce a TAN-rich solution with a low organic matter content, which is advantageous for nitrogen recovery applications such as in fertilizer production or nutrient recovery from wastewater treatment [40].

RO and NF membranes are highly effective at rejecting salts, which poses a challenge for TAN recovery due to their high rejection rates for both salts and TAN. To address this, we examined how varying the feed pH affected TAN rejection, noting that NH_4_^+^ predominates in acidic conditions, while NH_3_ becomes more prevalent in highly basic conditions. The study analyzed the filtration of three ammonium salts: NH_4_Cl, (NH_4_)_2_SO_4_, and NH_4_HCO_3_. According to the literature, NH_4_Cl and (NH_4_)_2_SO_4_ are the primary ammonium salts present in animal manure, especially in pig manure. NH_4_HCO_3_ becomes a more significant component after the anaerobic digestion of manure [41].

**Figure 3:**
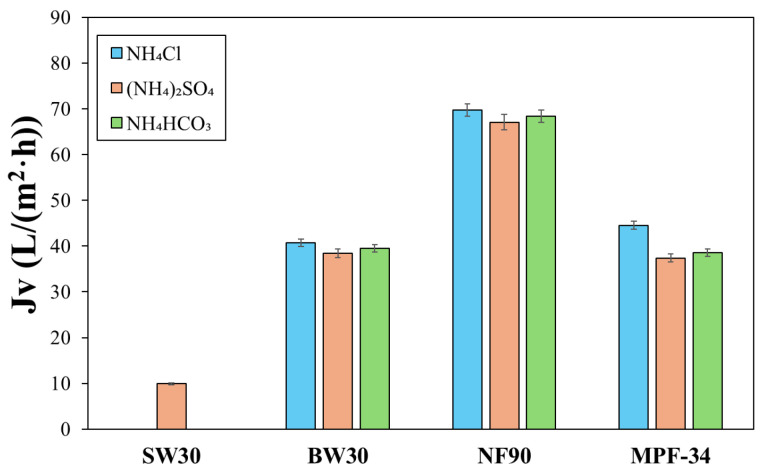
Mean permeate flux measured at 20 bar for the three ammoniums salts and the four membranes. The error bars were calculated with a 95% confidence limit.

We first analyzed the variation in permeate flux using three different ammonium salts and four membranes, across a range of feed pH values from 5.0 to 11.5. There was no statistically significant variance in the permeate flux with the feed pH. Figure 3 shows the mean permeate flux that was calculated from the data of all feed pH values, with the error bars based on a 95% confidence limit. The maximum observed error was 5.6%, which denoted the negligible effect of the feed pH on the permeate flux under the conditions evaluated. Similarly, the type of ammonium salt in the feed solution did not exhibit a significant influence on the permeate flux (Figure 3). The most notable difference was observed with the MPF-34 membrane, where the mean permeate flux was 44.5 L/(m^2^·h) for NH_4_Cl and 37.4 L/(m^2^·h) for (NH_4_)_2_SO_4_. Therefore, the mean permeate flux was calculated for each membrane, leading to the following values: 38.6 ± 0.9 L/(m^2^·h) for the BW30 membrane, 68.4 ± 0.8 L/(m^2^·h) for the NF90 membrane, and 40.2 ± 1.9 L/(m^2^·h) for the MPF-34 membrane. As expected, the SW30 membrane showed the lowest permeate flux among the evaluated membranes, with an average value of 9.89 ± 2.1 L/(m^2^·h). This value was recorded only with (NH_4_)_2_SO_4_ as it was ammonium salt used in the second stage of the hybrid system. No evidence has been found in the literature about the permeate flux with the three ammonium salts. Nevertheless, the permeate flux of those solutions has a similar trend to that observed with the NaCl solutions.

**Figure 4:**
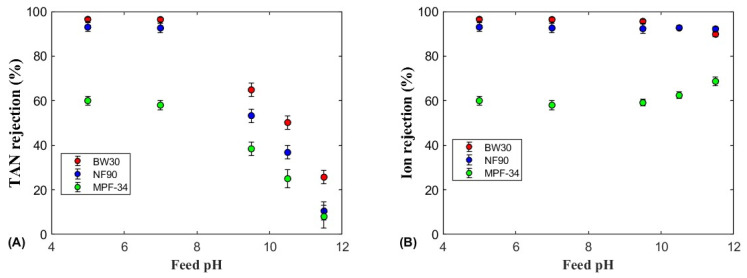
(**A**) TAN and (**B**) ion rejections for the NH_4_Cl feed solution as a function of the feed pH for the three membranes. The error bars were calculated with a 95% confidence limit.

**Figure 5:**
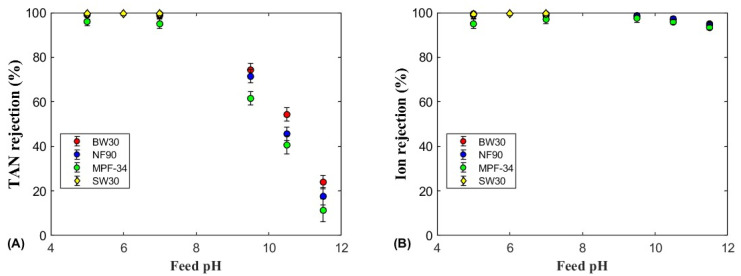
(**A**) TAN and (**B**) ion rejections for the (NH_4_)_2_SO_4_ feed solution as a function of the feed pH for the three membranes. The error bars were calculated with a 95% confidence limit.

**Figure 6:**
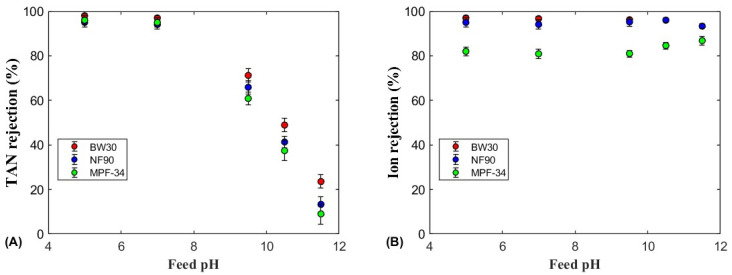
(**A**) TAN and (**B**) ion rejections for the NH_4_HCO_3_ feed solution as a function of the feed pH for the three membranes. The error bars were calculated with a 95% confidence limit.

The TAN and ion rejections as a function of the feed pH are shown in Figure 4, Figure 5 and Figure 6 for the four membranes (SW30, BW30, NF90, and MPF-34) and the three ammonium salts. All salts showed similar TAN rejection trends across the pH range, remaining relatively constant below pH 7, then decreasing almost linearly above pH 9.5. Among the salts, (NH_4_)_2_SO_4_ and NH_4_HCO_3_ achieved higher TAN rejection across all pH values, particularly with the BW30 and NF90 membranes. In contrast, NH_4_Cl showed lower rejection, especially with the MPF-34 membrane, which exhibited TAN rejection around 60% at pH 7.0 and 5.0. These results suggest that bigger anions such as sulfate and bicarbonate enhance the rejection of ammonium ions more effectively than the smaller monovalent chloride ions. Therefore, the size exclusion and Donnan potential are the main mechanisms to explain the high rejection values [32]. The MPF-34 membrane, with its larger MWCO, showed the lowest overall TAN rejection, particularly for NH_4_Cl, due to the reduced size exclusion as the membrane pore size are bigger [22].

In the pH range of 4–7, which is up to two units below the pKa of the ammonium–ammonia equilibrium (pKa = 9.24 at 25 °C), TAN rejection remained high and nearly independent of pH due to the very low concentration of uncharged ammonia. Ammonium sulfate showed the highest TAN rejection, exceeding 99% for the SW30, BW30, and NF90 membranes, with the SW30 membrane achieving a particularly high rejection rate of 99.5%. Kurama et al. (2002) showed that RO membranes can be used for removing ammonium anions from wastewater with a low TAN content [42]. Similarly, at an acidic pH, the MPF-34 membrane also showed high TAN rejection, although slightly lower at 96%. The ammonium cation, when paired with sulfate anions, showed high rejection values for the SW30, BW30, and NF90 membranes, primarily due to steric exclusion and the Donnan electric exclusion [10]. Then, these two exclusion mechanisms enhanced TAN rejection under acidic conditions, even for the MPF-34 membrane that has a larger MWCO. A similar trend was noted for NH_4_HCO_3_, although the maximum TAN rejection decreased slightly to 96–98% (Figure 6A). The slight reduction in TAN rejection may be attributed to the smaller size and lower charge of the bicarbonate anion compared to sulfate. Nonetheless, the BW30 and NF90 membranes continued to exhibit high rejection efficiencies for ammonium bicarbonate, while the MPF-34 membrane, though less effective, still achieved a rejection rate close to 98% under acidic conditions. The high TAN rejection observed with (NH_4_)_2_SO_4_ and NH_4_HCO_3_ can be attributed to the combined effects of ion size and electrostatic interactions between the sulfate and bicarbonate anions and the membrane surface. Additionally, the ammonium cation is rejected in the retentate stream as a result of the electroneutrality principle, which requires charge balance alongside the rejection of these anions [43].

At pH 9.5, which is close to the pKa of the ammonium–ammonia equilibrium, differences in TAN rejection were observed depending on the membrane and ammonium salt. For NH_4_Cl, a significant difference in rejection was observed for all the membranes, with BW30 showing the highest TAN rejection (69%), followed by NF90 (53%) and MPF-34 (39%). These results are consistent with the finding that BW30, with its denser structure, exhibits higher rejection rates for monovalent ions like NH_4_^+^. However, for (NH_4_)_2_SO_4_ and NH_4_HCO_3_, no significant differences in TAN rejection were noted among the membranes, with values ranging from 61% to 74% for (NH_4_)_2_SO_4_ and 60% to 72% for NH_4_HCO_3_.

At pH values above 9.5, where the equilibrium shifts towards free NH_3_ rather than NH_4_^+^ ions, the concentration of NH_3_ increased, leading to a decrease in TAN rejection for the three membranes and ammonium salts. This reduction in TAN rejection was due to the smaller molecular size and neutral charge of ammonia, which allowed it to permeate more easily through the membrane compared to the charged NH_4_^+^ ions. TAN rejection decreased for all three ammonium salts and the three membranes, following a nearly linear decline as the pH increased.

At pH 10.5, TAN rejection was more influenced by membrane type than by the specific ammonium salt. The BW30 membrane showed the highest TAN rejection for all three ammonium salts, with values of 54% for (NH_4_)_2_SO_4_, 50% for NH_4_HCO_3_, and 49% for NH_4_Cl. By contrast, the NF90 membrane showed moderate TAN rejection values with minor variations among the salts, while the MPF-34 membrane consistently showed the lowest rejection rates, particularly for NH_4_Cl (25%) and NH_4_HCO_3_ (37%). The lowest TAN rejection was observed at pH 11.5, with values dropping to approximately 10% for both the NF90 and MPF-34 membranes. The BW30 membrane exhibited a slightly higher rejection, ranging between 20% and 25%. The higher performance of BW30 can be attributed to its tighter pore size and higher rejection potential for both neutral and charged species, a feature reported in several studies on RO membranes. Limited research has examined NH_3_ rejection by nanofiltration membranes under basic pH conditions. One of the most relevant studies evaluated hydrolyzed human urine using BW30 and NF90 membranes in a dead-end configuration at pH values of 9 and 11.5 [25]. The results of that study demonstrated similar trends in NH_3_ rejection as a function of feed pH, with the NF90 membrane consistently exhibiting lower NH_3_ rejection compared to the BW30 membrane.

#### 3.2.2. Ion Rejection

Figure 4B, Figure 5B and Figure 6B show the ion rejection for the four membranes and three ammonium salts analyzed. For the SW30, BW30, and NF90 membranes, ion rejection remained high across a broad range of feed pH values below 9.5, with rejection values of 95% and 99.2%. This high ion rejection suggests that these membranes are effective at retaining anions, even in mildly alkaline conditions. Specifically, (NH_4_)_2_SO_4_ exhibited the highest ion rejection among the three salts due to sulfate anions being more effectively rejected via steric effects and the Donnan exclusion effect [44]. The enhanced ion rejection observed at acidic pH further supports the strong repulsion of sulfate anions, which is attributed to increased electrostatic interactions between the negatively charged membrane surface and the divalent sulfate ions [10,43].

As the feed pH increased, the ion rejection of these membranes showed a slight decrease, reaching 93%, due to the increase in the ammonia content of the feed solution. The presence of neutral solutes is known to reduce the ion rejection at high pH values [45].

By contrast, the MPF-34 membrane showed a different pattern of ion rejection, particularly with the NH_4_Cl and NH_4_HCO_3_ solutions. At pH values below 7, ion rejection remained relatively constant, with values of 60% for NH_4_Cl and 81% for NH_4_HCO_3_. This reduced performance can be attributed to the larger pore size of the MPF-34 membrane, which is more typical of loose nanofiltration and ultrafiltration membranes [46]. However, the ion rejection gradually improved with an increase in the feed pH. At pH 9–11.5, the ion rejection increased to 69% for NH_4_Cl and 87% for NH_4_HCO_3_. This improvement could be due to the membrane surface becoming more negatively charged as the functional groups deprotonate at higher pH values. Therefore, the ion rejection presented a minimum value at pH values between 7 and 8, which are close to the estimated IEP of the MPF-34 membrane [47]. At this point, the electrostatic interactions between the membrane surface and the solute are minimum, leading to a reduction in rejection. Studies such as those by Childress and Elimelech (2000) have emphasized the importance of the membrane surface charge in determining solute rejection, particularly near the IEP, where membranes exhibit their lowest rejection rates [48]. The minimum in ion rejection was not observed for the BW30 and NF90 membranes in the pH range 5–11 since they present an isoelectric point around 4 [49].

#### 3.2.3. The Role of Feed pH on TAN Rejection

The pH of the feed solution plays a critical role in influencing the stability and performance of the filtration membranes as it can significantly affect membrane integrity and permeability, particularly in long-term or continuous operation. The stability of a membrane at varying pH levels is determined largely by the chemical composition of the surface-active layer of the membrane. The BW30 and NF90 membranes, whose surface is composed of polyamide (Table 1), have a recommended operational pH range of 2 to 11, reflecting their susceptibility to hydrolysis and degradation in very alkaline conditions. In continuous operation, exceeding the upper pH limit (pH 11) may lead to membrane degradation and failure in overall performance due to the modification of the polyamide layer. Studies have shown that exposure to high pH values can cause irreversible damage to the polyamide structure, impacting flux and rejection performance [50,51]. Nevertheless, we conducted experiments in batch mode at a feed pH of 11.5 to evaluate TAN permeation, where all TAN is present as uncharged ammonia species. As previously mentioned, the maximum TAN permeation was observed at this pH. Moreover, several membrane coupons were utilized during the experiments, and no signs of membrane degradation were observed. Previous studies using the same membranes at a pH of 11.5 also reported no degradation over a short period [25,52]. By contrast, the MPF-34 membrane, which has an unknown surface layer composition, can tolerate a much broader pH range, extending up to pH 14. This superior pH tolerance allowed it to operate at pH 11.5 without compromised membrane performance, making it suitable for processes that require exposure to highly alkaline environments.

**Figure 7:**
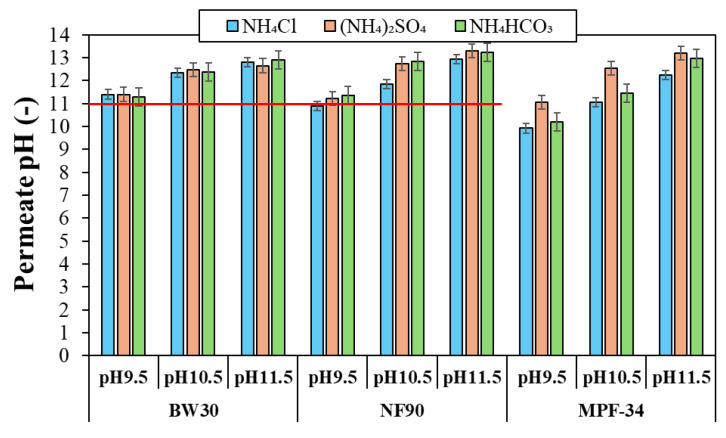
Permeate pH as a function of the feed pH and ammonium salt for the three membranes tested. The red line corresponds to the maximum operational pH of the BW30 and NF90 membranes. The error bars were calculated with a 95% confidence limit.

Figure 7 shows the permeate pH values for the three ammonium salts at alkaline feed pH values for the BW30, NF90, and MPF-34 membranes. Minimal differences in the permeate pH were observed among the ammonium salts, suggesting that the type of ammonium salt has a slight effect on pH behavior under the tested conditions. Although the permeate stream showed slightly higher pH values for NH_4_HCO_3_, these variations were not significant. As the feed pH increased from 9.5 to 11.5, the permeate pH generally increased, approaching or exceeding pH 11. The high pH levels observed in the permeate are not solely due to NH_3_ permeation but may also result from the co-transport of OH^−^, together with Na^+^ or NH_4_^+^, to maintain charge balance in both retentate and permeate streams. For both the BW30 and NF90 membranes, the pH of the permeate stream was relatively similar for the three ammonium salts at each feed pH value, with mean values of 11.3, 12.4, and 13.0 for the feed solution pH values of 9.5, 10.5, and 11.5, respectively. These pH values of the permeate exceeded the pH limit recommended by the manufacturers. Since the recommended pH value of 11 applies to the feed and retentate streams, it is likely that the pH within the membrane matrix is lower than that in the permeate stream but still higher than 11. Thus, it can be inferred that the maximum operational feed pH in the continuous mode for both the BW30 and NF90 membranes should be around 9.5 to avoid potential membrane degradation. Meanwhile, the MPF-34 membrane showed more variation in permeate pH depending on the feed solution pH and the specific ammonium salt used. Particularly, (NH_4_)_2_SO_4_ resulted in higher permeate pH values compared to NH_4_Cl and NH_4_HCO_3_ with the three feed pH values. This behavior is likely related to the higher ion rejection observed for (NH_4_)_2_SO_4_, which approaches unity, in contrast to the lower ion rejection values obtained for NH_4_Cl and NH_4_HCO_3_. The high rejection of (NH_4_)_2_SO_4_ is likely due to size exclusion and Donnan-based charge exclusion effects, driven by the interaction of divalent sulfate anions with the MPF-34 membrane. This leads to a more negatively charged retentate stream, which may enhance the diffusion of hydroxide ions (OH^−^) toward the permeate stream, thereby increasing the permeate pH in the case of (NH_4_)_2_SO_4_. Notably, all measured permeate pH values for the MPF-34 membrane remained below pH 14, which is within the membrane’s recommended operational limit.

### 3.3. Effect of Organic Matter on TAN Rejection

The impact of organic matter on membrane performance was evaluated to better understand its effect on TAN rejection under conditions that more closely resemble real livestock wastewater. For example, pig manure filtrates, following filtration and ultrafiltration pretreatment, typically contain low-molecular-weight organic compounds capable of passing through ultrafiltration membranes due to their size and structural characteristics [53]. The resulting ultrafiltration permeate is subsequently treated using RO or NF membranes for further purification. The composition of these small organic molecules can vary depending on the type of livestock waste. In this study, glucose, a common carbohydrate, and glycine, an amino acid, were selected as model compounds to represent the small organic molecules.

**Figure 8:**
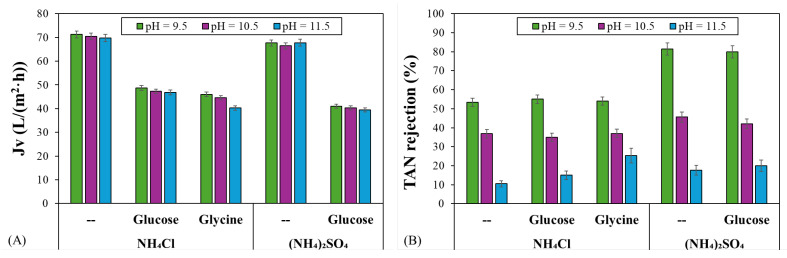
(**A**) Permeate flux and (**B**) TAN rejection for the NF90 membrane with NH_4_Cl solution and (NH_4_)_2_SO_4_ solution as a function of the feed pH in the presence of glucose or glycine. The error bars were calculated with a 95% confidence limit.

Figure 8 shows the permeate flux and TAN rejection by the NF90 membrane as a function of the feed pH. The data incorporated different feed solutions: 0.2 M NH_4_Cl with 0.1 M glucose, 0.2 M NH_4_Cl with 0.1 M glycine, and 0.2 M (NH_4_)_2_SO_4_ with 0.1 M glucose. In addition, the permeate flux and TAN rejection for NH_4_Cl and (NH_4_)_2_SO_4_ without organic molecules were outlined for comparison, aiming to clarify the influence of organic compounds on membrane performance.

The presence of glucose or glycine in the NH_4_Cl and (NH_4_)_2_SO_4_ feed solutions significantly reduced the permeate flux for the three feed pH values tested, likely due to the increased feed osmotic pressure as a result of the presence of these organic solutes. For instance, the permeate flux for NH_4_Cl at pH 10.5 decreased by 33% in the presence of glucose and by 37% in the presence of glycine. The reduction for (NH_4_)_2_SO_4_ at this pH reached 39%. The higher permeate flux reduction of 43% was noted for NH_4_Cl and glycine at pH 11.5. These findings indicate that while the type of the organic solute impacted flux reduction, variations in the feed pH within the tested range did not significantly alter the permeate flux. Furthermore, the rejection of glucose and glycine was evaluated as a function of feed pH, with results indicating consistently high rejection rates for both organic solutes, regardless of the feed pH. The average rejection values were 99.1% ± 0.4% for glucose and 98.7% ± 0.3% for glycine, suggesting that the NF90 membrane effectively retained these organic molecules. These results align with prior findings on the rejection of similar organic solutes under pH variations [22,54,55].

As discussed above, TAN rejection decreased significantly as the feed pH increased, leading to high ammonia permeation. This trend was observed even in the presence of glucose and glycine, where TAN rejection similarly decreased with a rising feed pH. At pH 9.5 and 10.5, the presence of glucose and glycine in the NH_4_Cl and (NH_4_)_2_SO_4_ feed solutions did not significantly modify TAN rejection. However, a slight increase in TAN rejection was observed at pH 11.5, particularly in the presence of glycine. This increase may be attributed to the negative charge of both glucose and glycine under highly alkaline conditions. For instance, glucose, with a pKa of 12.1, and glycine, with pKa values of 2.37 and 9.60, become negatively charged as the solution pH exceeds 10.5 [47,56]. The increased negative charge of these solutes at high pH values may modify the ammonium/ammonia equilibrium by reducing the concentration of free ammonia in the feed solution, leading to both higher TAN rejection and a lower permeate flux, especially in very alkaline conditions.

### 3.4. Evaluation of Hybrid Systems

**Figure 9:**
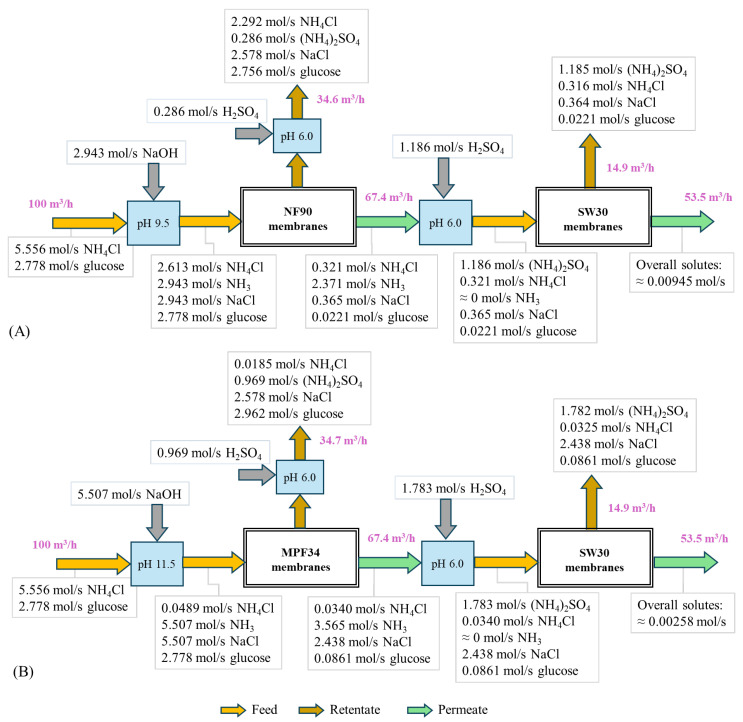
Schematic representation of the hybrid membrane systems analyzed with the solute molar flows, which were calculated from the mass balance. (**A**) The NF90–SW30 system and (**B**) the MPF-34–SW30 system.

We analyzed a continuous TAN recovery process using a hybrid NF and RO system in a double-pass configuration, which enhanced separation efficiency and nutrient recovery [57]. Each stage in the process included a homogenization tank to adjust the pH and a membrane rack. A simplified schematic of the system is provided in Figure 9, while the complete process flow sheet and model equations are shown in the Supplementary Materials. In the first stage, the ammonium ions in the feed are converted into ammonia by adding NaOH to increase the pH. This stage employs an NF membrane rack, and a portion of the retentate stream is recycled back into the feed to enhance overall process efficiency [58]. In the second stage, sulfuric acid is introduced to reduce the pH of the permeate stream from the first stage to convert the ammonia into ammonium sulfate. This stage utilizes an SW30 RO membrane rack to further purify the permeate, with a partial recycling of the permeate stream back to the feed stream to improve the efficiency of the system.

This section aimed to evaluate TAN and water recovery within a continuous conceptual process using the NF90 and MPF-34 membranes in the first stage. Mass balance calculations were applied to determine volumetric flows and molar concentrations of all the solutes, incorporating experimentally obtained solute rejection values. To ensure consistent solute concentrations, electroneutrality was enforced for each stream by accounting for all relevant ions. For example, in the feed stream of the first stage, the ions considered included NH_4_^+^, H^+^, Na^+^, OH^−^, and Cl^−^. The set of equations was solved using defined input parameters specific to each system under evaluation. The feed flow rate of the system was fixed at 100 m^3^/h, using 0.2 M NH_4_Cl and 0.1 M glucose. Additionally, both the retentate stream recycle ratio and the water recovery in the first stage were fixed at 50%, based on the operational range of the NF90 membrane module for a volumetric feed flow. Under these conditions, the first stage achieved an overall water recovery of 66.6%. In the second stage, a permeate stream recycle ratio of 10% and a water recovery rate of 80% were established. The required amounts of NaOH and H_2_SO_4_ for the system depended on the operating pH of each stage. For the NF90 and MPF-34 membranes in the first stage, the pH was adjusted to 9.5 and 11.5, respectively. Higher pH values improve the TAN separation efficiency with the MPF-34 membrane due to its resistance to alkaline conditions [47]. In the second stage, the pH was maintained consistently at 6.0 for both membrane configurations.

Figure 9 shows a schematic representation of the continuous operation, illustrating the volumetric flow rates and molar solute fluxes for both the hybrid NF90–SW30 and MPF-34–SW30 membrane systems. Both systems achieved a water recovery rate of 53.5%, yielding permeate streams of high quality. The TAN concentrations in the final permeate were 4.80 mg/L for the NF90–SW30 system and 6.37 mg/L for the MPF-34–SW30 system. In terms of nitrogen content, it represents 3.73 and 4.95 mg/L, respectively, which are much lower values than fixed by the reuse following the Spanish regulation [59]. Additionally, low contents of total dissolved solids (TDSs) were observed in the permeate, with concentrations of 14 mg/L for the NF90–SW30 system and 28 mg/L for the MPF-34–SW30 system. These nitrogen and TDS levels align with some water reuse goals [60]. For instance, for aquifer recharge, the maximum acceptable value of the total nitrogen content is 10 mg/L and of the total suspended solids is 35 mg/L [59]. TAN recovery was evaluated as the molar flow ratio between the second-stage retentate and the system feed. The results indicated a difference in TAN recovery due to pH variations in the first stage, primarily due to the higher pH conditions applied in the first stage of the MPF-34–SW30 system. Especially, TAN recovery in the NF90–SW30 system was 48.3%, with a TAN concentration of 11.7 g/L in the second-stage retentate. By contrast, the MPF-34–SW30 system enhanced TAN recovery to 64.8%, with a retentate TAN concentration of 15.6 g/L. Consequently, the final concentrations of nitrogen in the two retentate streams were 0.9% N and 1.22% N for the NF90–SW30 and MPF-34–SW30 system, respectively. Moreover, the two streams contained minimal organic matter content, as indicated by a glucose molar fraction of less than 2%. Only a limited number of studies have analyzed hybrid NF–RO systems for nitrogen recovery. One such study investigated urea recovery from human urine with a feed nitrogen concentration of 5.7 g/L, reporting an overall water recovery of 80% and a urea recovery of 32.7% with high purity [61].

Liquid ammonium sulfate is a nitrogen fertilizer with an acidifying effect, ideal for vegetables and gardens. Its nitrogen is entirely in ammoniacal form, providing a steady and long-lasting effect [62,63]. Due to its acidifying action, it is recommended for calcareous (lime-rich) soils. Commercially available as a concentrated solution containing 8% nitrogen, it must be diluted to approximately 0.16% N for solid irrigation, requiring over 50-fold dilution and resulting in high clean water consumption [64]. In this context, our two proposed hybrid systems yield final fertilizers with lower nitrogen content than the concentrated commercial product, offering a significant reduction in clean water usage before their reuse in irrigation.

Ammonium sulfate is commonly produced via direct synthesis, where NH_3_ reacts with H_2_SO_4_ in stoichiometric proportions. The process is straightforward and efficient, yielding a solution of ammonium sulfate that is subsequently crystallized into a solid product [65]. Our approach follows a similar principle but replaces the traditional reactor with membrane modules. The key distinction is the ammonia source: instead of relying on ammonia from the energy-intensive Haber–Bosch process, we utilize recovered ammonia from livestock waste. This offers environmental benefits and reduces dependence on synthetic fertilizers, following the Circular Economy Action Plan [66]. The European Union expects that fertilizers coming from nutrient recovery will replace up to 30% of the inorganic fertilizers currently used [67].

A Life Cycle Assessment (LCA) could be undertaken in future research to evaluate the environmental performance of liquid fertilizer production. This analysis would involve defining multiple scenarios to compare potential improvement strategies and assess their environmental impacts [3,68]. Key factors would include electricity use by pressure-generating pumps and membrane manufacturing and replacement, as well as other operational inputs. Additional considerations, such as raw material sourcing, water consumption, and waste generation, could also be incorporated. A LCA would offer valuable insights for optimizing the process and improving its sustainability, providing a strong basis for further research and development.

In this study, however, we focus solely on evaluating chemical consumption costs as this is a key factor influencing TAN rejection and recovery. The hybrid MPF-34–SW30 system demonstrated greater suitability for TAN recovery, although it had a higher chemical consumption, as evidenced by the molar flow rates of NaOH and H_2_SO_4_ used for the pH adjustments (Figure 9). The total consumption of the two reagents, which was quantified as the mass flow rate ratio between the chemicals consumed and the (NH_4_)_2_SO_4_ generated by the system, was 0.753 kg/kg and 0.922 kg/kg for the NF90–SW30 system and 0.936 kg/kg and 1.146 kg/kg for the MPF-34–SW30 system, respectively. The increased acid and base consumption in the MPF-34–SW30 system was attributed to its higher operating pH (set between 9.5 and 11.5) due to the greater tolerance of the MPF-34 membrane for alkaline conditions, as well as the enhanced TAN ion transport through the membrane [69]. This also has implications for the applied pressure on the membrane racks as the MPF-34 membrane has a lower water permeation rate and a higher TAN permeation rate [70]. Although both hybrid systems showed effective TAN recovery, the MPF-34–SW30 system exhibited higher chemical and energy consumption, thereby increasing overall costs. As a preliminary assessment, the chemical consumption costs were calculated using the following prices: 0.3 EUR/kg NaOH (50%) and 0.14 EUR/kg H_2_SO_4_ (96%) [71]. The resulting overall chemical costs were 0.586 EUR/kg of (NH_4_)_2_SO_4_ generated for the NF90–SW30 system and 0.729 EUR/kg of (NH_4_)_2_SO_4_ generated for the MPF-34–SW30 system, reflecting a higher cost of 24.4% for the latter. The determination of the optimal cost-effective system for yielding a reusable fertilizer stream requires a comprehensive economic assessment, which will be performed and disseminated in future research. Nevertheless, it is important to acknowledge that the main value of nutrient recovery for fertilizer production extends beyond conventional economic parameters.

## 4. Conclusions

TAN rejection was evaluated in two RO and two NF membranes as a function of the feed pH and with different ammonium salts. The results showed that both membrane type and feed pH significantly influenced TAN rejection, with acidic conditions generally enhancing rejection efficiency. The size of the ammonium salt also influences TAN rejection. Salts containing smaller anions, such as chloride, exhibited lower rejection due to reduced steric hindrance and weaker electrostatic interactions with the membrane.

Among the tested membranes, BW30 and NF90 performed well under acidic to neutral pH, while the MPF-34 membrane showed lower overall rejection, particularly at high pH with NH_4_Cl. Interestingly, MPF-34 exhibited an unexpectedly low permeate flux for an NF membrane (comparable to that of the BW30 RO membrane) likely due to its high active layer thickness. At pH 11.5, the lowest TAN rejection (around 10%) was observed, indicating high permeability of uncharged NH_3_.

Two theoretical hybrid membrane systems were also evaluated: NF90–SW30 and MPF-34–SW30. In both configurations, the first-stage NF membrane operated under alkaline conditions to promote NH_3_ permeation, followed by the SW30 RO membrane at pH 6.5 to recover TAN as (NH_4_)_2_SO_4_, achieving over 99.6% rejection. TAN recovery reached 55% in the NF90–SW30 system (first-stage pH 9.5) and 66% in the MPF-34–SW30 system (first-stage pH 11.5). Both systems produced concentrated streams with nitrogen contents of 0.9% and 1.2%, respectively, suitable for direct use as liquid fertilizer in agricultural irrigation. Although the hybrid systems require considerable chemical and energy inputs, they represent a novel and promising approach for efficient TAN recovery from high-content wastewater. This membrane-based strategy supports sustainable resource management and contributes to circular economy objectives by enabling direct fertilizer production from wastewater-derived nutrient.

## Figures and Tables

**Table 1:** Key properties and performance specifications as specified by the manufacturer.

Membrane	Material of the Skin Layer	MgSO_4_ Rejection (%)	NaCl Rejection (%)	Permeability Coefficient (L/(m^2^·h·bar))	Molecular Weight Cut-Off (Da)	Membrane Isoelectric Point (IEP)	Operation pH
SW30	Fully aromatic polyamide		99.7 ^(a)^	0.778 ^(a)^	N/A	~3.8 [29]	2–11
BW30	Fully aromatic polyamide		99.1 ^(b)^	4.79 ^(b)^	<100	~4.2 [29]	2–11
NF90	Fully aromatic polyamide	98.7 ^(c)^		8.92 ^(c)^	~100	~4.0 [29]	2–11
MPF-34	Proprietary		35 ^(d)^	2.00 ^(d)^	~200	~6.5 [22]	0–14

^(a)^ Test conditions: 32.0 g/L NaCl, 55 bar, 25 °C, 15% recovery. ^(b)^ Test conditions: 2.0 g/L NaCl, 10.3 bar, 25 °C, 15% recovery. ^(c)^ Test conditions: 2.0 g/L MgSO_4_, 4.8 bar, 25 °C, 15% recovery. ^(d)^ Test conditions: 50.0 g/L NaCl, 30 bar, 30 °C, 15% recovery.

## Data Availability

The data supporting this study’s findings are available upon request from the corresponding author.

## Supplementary Materials

The following supporting information can be downloaded at: https://www.mdpi.com/article/10.3390/polym17121696/s1. Figure S1: The process flow sheet for TAN recovery in a continuous operating mode, and model equations for the hybrid system.
