# Supplementary material for: Study of Total Ammoniacal Nitrogen Recovery Using Polymeric Thin-Film Composite Membranes for Continuous Operation of a Hybrid Membrane System
Source: Polymers (Basel). 2025 Jun 18;17(12):1696. doi: 10.3390/polym17121696 (PMC12196730; doi:10.3390/polym17121696)
Supplement: Supplementary file 1 [file polymers-17-01696-s001.zip › polymers-3666917-supplementary.pdf]

Supplementary Material

Study of Total Ammoniacal Nitrogen Recovery Using Polymeric Thin-Film Composite Membranes for Continuous Operation of a Hybrid Membrane System

Shirin Shahgodari, Joan Llorens and Jordi Labanda*

Department of Chemical Engineering and Analytical Chemistry, University of Barcelona, Martí i Franquès 1, 08028 Barcelona, Spain

*Corresponding author e-mail: jlabanda@ub.edu

This supplementary material detailed the hybrid nanofiltration-reverse osmosis system proposed for TAN recovery in a continuous operating mode. The detailed process flow sheet is shown in Figure S1, which is composed by two stages: (i) ammonia recovery using nanofiltration membranes and (ii) ammonium recovery using reverse osmosis membranes.

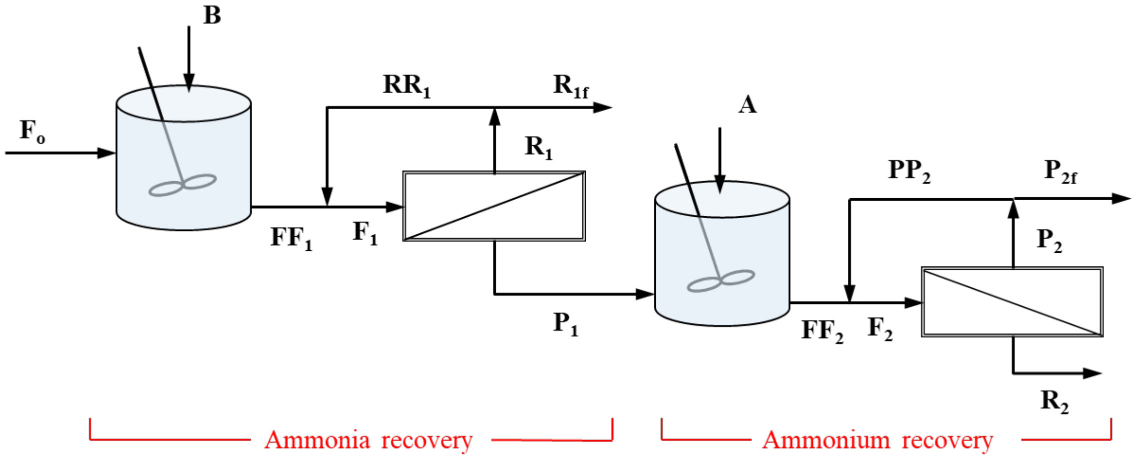

Figure S1. The process flow sheet for TAN recovery in a continuous operating mode. Stream B represents the addition of NaOH, while stream A corresponds to the addition of H_2SO_4.

Model equations for the hybrid system

The global mass balances, the recirculation ratios and the water recovery of the two sections are the following:

$$F + B = FF_1$$
$$FF_1 + RR_1 = F_1$$
$$F_1 = R_1 + P_1$$
$$R_1 = RR_1 + R_{1f}$$
$$r_1 = \frac{RR_1}{R_1}$$
$$\theta_1 = \frac{P_1}{F_1}$$
$$P_1 + A = FF_2$$
$$FF_2 + PP_2 = F_2$$
$$F_2 = R_2 + P_2$$
$$P_2 = PP_2 + P_{2f}$$
$$r_2 = \frac{PP_2}{P_2}$$
$$\theta_2 = \frac{P_2}{F_2}$$

The nitrogen (N) mass balances and the nitrogen rejections ($\Re_{1N}$ *and* $\Re_{2N}$) of the two stages are the following:

$$F \cdot C_{FN} = FF_1 \cdot C_{FF1N}$$
$$FF_1 \cdot C_{FF1N} + RR_1 \cdot C_{R1N} = F_1 \cdot C_{F1N}$$
$$F_1 \cdot C_{F1N} = R_1 \cdot C_{R1N} + P_1 \cdot C_{P1N}$$
$$\Re_{1N} = 1 - \frac{C_{P1N}}{C_{R1N}}$$
$$P_1 \cdot C_{P1N} = FF_2 \cdot C_{FF2N}$$
$$FF_2 \cdot C_{FF2N} + PP_2 \cdot C_{P2N} = F_2 \cdot C_{F2N}$$
$$F_2 \cdot C_{F2N} = R_2 \cdot C_{R2N} + P_2 \cdot C_{P2N}$$
$$\Re_{2N} = 1 - \frac{C_{P2N}}{C_{R2N}}$$

The sodium ion (Na) mass balances and their rejections of the two sections are the following:

$$B \cdot C_B = FF_1 \cdot C_{FF1Na}$$
$$FF_1 \cdot C_{FF1Na} + RR_1 \cdot C_{R1Na} = F_1 \cdot C_{F1Na}$$
$$F_1 \cdot C_{F1Na} = R_1 \cdot C_{R1Na} + P_1 \cdot C_{P1Na}$$
$$\Re_{1Na} = 1 - \frac{C_{P1Na}}{C_{R1Na}}$$
$$P_1 \cdot C_{P1Na} = FF_2 \cdot C_{FF2Na}$$

$$FF_2 \cdot C_{FF2Na} + PP_2 \cdot C_{P2Na} = F_2 \cdot C_{F2Na}$$
$$F_2 \cdot C_{F2Na} = R_2 \cdot C_{R2Na} + P_2 \cdot C_{P2Na}$$
$$\Re_{2Na} = 1 - \frac{C_{P2Na}}{C_{R2Na}}$$

where C_B is the concentration of NaOH added to adjust the pH in the stream FF1.

The mass balances and rejections for the chloride (C) of the two sections are the following are the following:

$$F \cdot C_{FC} = FF_1 \cdot C_{FF1C}$$
$$FF_1 \cdot C_{FF1C} + RR_1 \cdot C_{R1C} = F_1 \cdot C_{F1C}$$
$$F_1 \cdot C_{F1C} = R_1 \cdot C_{R1C} + P_1 \cdot C_{P1C}$$
$$\Re_{1C} = 1 - \frac{C_{P1C}}{C_{R1C}}$$
$$P_1 \cdot C_{P1C} = FF_2 \cdot C_{FF2C}$$
$$FF_2 \cdot C_{FF2C} + PP_2 \cdot C_{P2C} = F_2 \cdot C_{F2C}$$
$$F_2 \cdot C_{F2C} = R_2 \cdot C_{R2C} + P_2 \cdot C_{P2C}$$
$$\Re_{2C} = 1 - \frac{C_{P2C}}{C_{R2C}}$$

and the sulphate anion (S) mas balances and rejection ($\Re_{2S}$) in the second stage are the following:

$$A \cdot C_A = FF_2 \cdot C_{FF2S}$$
$$FF_2 \cdot C_{FF2S} + PP_2 \cdot C_{P2S} = F_2 \cdot C_{F2S}$$
$$F_2 \cdot C_{F2S} = R_2 \cdot C_{R2S} + P_2 \cdot C_{P2S}$$
$$\Re_{2S} = 1 - \frac{C_{P2S}}{C_{R2S}}$$

where C_A is the concentration of H_2SO_4 added to adjust the pH in the stream FF2.

The model is completed with the electroneutrality condition in the streams FF1, F1, R1, P1, FF2, F2, R2 and P2. As an example, the electroneutrality condition in the stream FF1 and FF2 for chloride anion are the following:

$$C_{FF1NH4} + C_{FF1H} + C_{FF1Na} = C_{FF1OH} + C_{FF1C}$$

$$C_{FF2NH4} + C_{FF2H} + C_{FF2Na} = C_{FF2OH} + C_{FF2C} + 2 \cdot C_{FF2S}$$

where the subscripts H and OH denote the concentrations of protons and hydroxide ions, respectively. The proton concentration can be readily determined from the pH value, while the hydroxide concentration can be

calculated using the self-ionization constant of water. The ammonium ion concentrations are calculated from the corresponding acid-base equilibrium, K_{aN}, as follows:

$$C_{FF1NH4} = \frac{C_{FF1N} \cdot C_{FF1H}}{C_{FF1H} + K_{aN}} \quad ; \quad C_{FF2NH4} = \frac{C_{FF2N} \cdot C_{FF2H}}{C_{FF2H} + K_{aN}}$$

The model is based on 48 equations and 66 variables. To solve the system, it has to be established the value of 18 variables. Some of them are determined from experimental results. For instance, F, C_{FN}, C_{FC}, $\Re_{1N}$, $\Re_{2N}$, $\Re_{1Na}$, $\Re_{2Na}$, $\Re_{1C}$, $\Re_{2C}$ and $\Re_{2S}$. Other variables can be fixed to analyze the effect of those variables on the final calculations. For instance, A, B, r_1, θ_1, r_2, θ_2, pH_{FF1} and pH_{FF2}. Therefore, the model is based on 48 equations and 48 unknown variables.
